# Emerging Challenges and Threats for Dental Health Care Sector Attributable to COVID-19: Tale of a Developing Country

**DOI:** 10.1177/1010539520932708

**Published:** 2020-06-04

**Authors:** Asma Sadruddin Pethani, Raheel Rehman Allana, Mehmood Hussain

**Affiliations:** 1 Aga Khan University, Karachi, Pakistan; 2 Dow University of Health Sciences, Karachi, Pakistan; 3 Hamdard University, Karachi, Pakistan

**Keywords:** cross-infection, dentistry, microorganisms, outbreak preparedness, challenges

## Abstract

Prevention and control of infection in dentistry is an essential matter that has gained immense attention in recent years. There exist a lack of scientific data about the cross-transmission and its associated risk, especially in the dental health care setting of Pakistan. This article will evaluate the emerging challenges and threats for the dental community in Pakistan attributable to the COVID-19 outbreak. There is a significant knowledge gap regarding the state- and institutional-level of infection prevention and control (IPC) policies and practices. In addition, not much is known about the development, implementation, and monitoring of IPC policies and its challenges.

## Introduction

Globally more than 3.5 million cases of COVID-19 have been reported (Source: World Health Organization Situation Report No. 107). With time, cases in China reduced but the epidemic started to spread rapidly across the world. Pakistan is currently in the midst of this epidemic, which has led authorities to impose social distancing to reduce its spread. It would not be an underestimation to claim that COVID-19 has created havoc across the globe.^1^ It has affected all industries including dental. In recent years, the global burden of oral health disease is 2-fold both for developed and developing nations.^2^ It is of huge concern that dental care services are not readily accessible to most of the population due to the expensive nature of treatment modalities. In most of the developing countries, the situation is further impacted by the fact that dental care is not well regulated and not even considered as one of the essential components of the primary health care system.^2,3^ On top of that, in the state of a recent global pandemic of COVID-19, it is questionable whether the dental practitioners would be consistently utilizing safe infection control practices.

## Infection Control Practices at Dental Facilities

### Risk of Infection Transmission at Dental Facilities

During the dental procedures, the use of ultrasonic instruments and high-speed hand-pieces is very common. These instruments aerosolize oral secretions such as saliva and blood into the surroundings. Due to the unique nature of dental procedures, which generates large number of aerosols and droplets, the usual standardized protective measures followed by the dental care workers will not suffice for preventing the spread of COVID-19, especially when the patient is symptom-free, unaware about the disease status, or falsifying infection history. Therefore, the containment of the propagation of the virus would be nearly impossible.^4^

### Gap Analysis of Coherent Infection Control Practices

**Figure 1.**
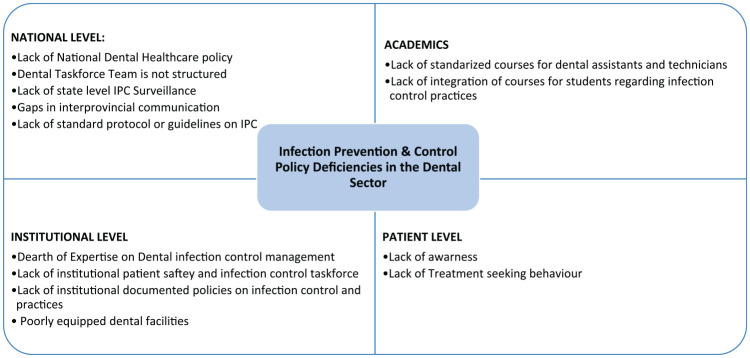
Cause and effect diagram for understanding infection prevention and control (IPC) policy deficiency in the dental sector.

The oral cavity is the natural habitat for heaps of pathogens and opportunistic microorganisms. These have imposed an immense risk for cross-contamination and infections. In dental practice, the risk of exposure to microorganisms further intensified due to the open and invasive nature of management regimens. There is evidence that in dentistry, hepatitis B virus is a real menace for cross-infection.^5^ However, in our context, there is a huge gap with the scientific data for viral and bacterial infection and transmission in dental practices. The cross-infection is underreported in the dental literature. Lack of adherence to standard precautions remains a challenging obstacle. The common examples for noncompliance with the infection control practices in dentistry are never sterilizing dental hand-pieces; never disinfecting impressions/casts; and the occurrence of dried blood on dental instruments such as on hand-mirror heads, on the dental chair, and dental burs.^6^ Furthermore, it has been exacerbated with the lack of hand hygiene practices while switching patients due to carefree attitudes, never disinfecting the work environment between procedures, lack of provision of protective gears, nonavailability of alcohol-based hand rub, improper dental waste management, and lack of safe water supply in dental units.^6^ For further understanding of the convolutions, a cause and effect diagram is shown in Figure 1. With ineffective infection control practices, it would be challenging to protect dental health workers and patients from the transmission of preventable infections during and after pandemic situation. Therefore, many of the academic institutes and private clinics have shut down their practices or limited to emergency services due to the highly contagious nature of COVID-19 and unavailability of necessary personal protective equipment such as full-sleeved impervious gowns, surgical gloves, respirators, head caps, and face shields.

## Way Forward

An effective infection control and prevention program can only be attained when there is a systematic approach for implementing all the vital elements in the dental sector. These core components should be implemented both at nationwide hospitals affiliated with dental institutes and private dental care facilities. The following actions are proposed.

### Member States/Regulatory Bodies

1. A state-level task force should be formed with individuals trained in infection prevention. They would be responsible for developing documented countrywide evidence-based policy procedures and guidelines. It should include guidelines for hand hygiene, use of personal protective equipment, respiratory hygiene/cough etiquette, sharps safety, safe injection practices, instruments sterilization, disinfected environmental surfaces, and dental unit water quality.
2. State-level infection prevention and control key performing indicators should be defined, as well as a mechanism that would allow regular monitoring and evaluation.
3. Introducing regulatory mechanisms for accreditation of dental health care facilities to ensure compliance with infection control standards.

### Institutional Administrative Measure

1. Agreed upon international and national policies should be tailored for academic institutions and should be gauged at defined periodic intervals.
2. The hiring of an infection prevention coordinator to ensure compliance of infection control standards.
3. Development and implementation of evidence-based triage process to prioritize and manage infectious patients.

### Infection Prevention Education and Training

1. Mandatory on-job infection control training of dental providers and students.
2. Identify standard training component for infection control and prevention such as precautionary measures, mode of transmission, and its prevention.

## Conclusion

There exists a significant gap in the development and implementation of infection control and patient safety policies at dental health care facilities. Without the appropriate infection prevention practices, dental facilities will be the permanent home for transmission of viruses and bacteria within the facilities and to the community, which will further impart resistance. In the past, the understandings from the epidemics such as severe acute respiratory syndrome (SARS), H1N1, Ebola, and the recent COVID-19 demonstrate that health care facilities can amplify newer microorganisms and diseases to the patients, health care workers, and community if there will be no appropriate and effective dental infection prevention and control policies in place.
